# An *in vivo* crosslinking system for identifying mycobacterial protein–protein interactions

**DOI:** 10.1016/j.mimet.2014.07.012

**Published:** 2014-10

**Authors:** Kathryn E.A. Lougheed, Mark H. Bennett, Huw D. Williams

**Affiliations:** Department of Life Sciences, Imperial College London, SW7 2AZ, United Kingdom

**Keywords:** *Mycobacterium tuberculosis*, Tandem affinity purification, Formaldehyde crosslinking, Pyruvate dehydrogenase, AceE

## Abstract

The analysis of protein–protein interactions in *Mycobacterium tuberculosis* has the potential to shed light on the functions of the large number of predicted open-reading frames annotated as conserved hypothetical proteins. We have developed a formaldehyde crosslinking system to detect *in vivo* interactions in mycobacteria. Our Gateway-adapted vector system uses three promoter strengths, including constitutive and regulatable versions, for the expression of target proteins with either an N- or C-terminal His–Strep–Strep tag. Tandem affinity purification using the His- and Strep-tags is well-suited to the isolation of protein complexes with a high purity and no detectable background. We have validated this approach using the well-described pyruvate dehydrogenase complex.

## Highlights

• We have developed vectors for *in vivo* cross linking studies in mycobacteria.
• The Gateway-adapted vector system provides three promoter strengths.
• Proteins can be expressed with an N- or C-terminal His–Strep–Strep tag.
• The system was validated using the pyruvate dehydrogenase complex.

## 1 Introduction

*Mycobacterium tuberculosis* (*Mtb*) remains a serious threat to global health, with latent infections representing a huge burden of potentially infectious bacteria. An improved understanding of the molecular basis of its unique ability to survive long periods of non-growth in the human host is one of the key challenges in the post-genomic era. However, a gene function is so far undescribed for a large number of the predicted *Mtb* ORFs (Cole et al., 1998). Our past experience in using genetic approaches to investigate the function of conserved hypothetical proteins has proven ill-suited in certain cases (Hingley-Wilson et al., 2010), likely due to the large degree of redundancy present within vital *Mtb* pathways.

A number of techniques are available for studying protein interactions in mycobacteria, such as the Split-Trp (O'Hare et al., 2008) and mycobacterial protein fragment complementation (M-PFC) system (Singh et al., 2006). While the M-PFC system has been used to screen for interactions with a bait protein through the use of library of prey plasmids, this is labor-intensive and relies on the expression of both bait and prey with a large N- or C-terminal tag which is not suitable for all proteins. Two-hybrid systems are also prone to high numbers of false positive interactions (Battesti and Bouveret, 2012).

The use of tandem affinity purification (TAP) tags can allow proteins to be expressed *in vivo* and purified with their associated partners. However, this has a disadvantage in that non-specific interactions are often detected and weak or transient interactions can be missed. Formaldehyde crosslinking has therefore been previously used to fix interactions prior to more stringent purification methodologies that yield highly-pure protein complexes (Herzberg et al., 2007). Formaldehyde is capable of penetrating the unusually impermeable mycobacterial cell wall and crosslinks proteins within around 2 Å of each other. One potential disadvantage in the sensitivity of this method is that high-level expression of proteins under non-native conditions could lead to the identification of biologically irrelevant interactions. In addition, some proteins are toxic when expressed at high levels. Therefore, it is desirable to be able to vary expression levels to better reproduce the *in vivo* conditions under which natural protein–protein interactions occur. Here we report the construction of a series of Gateway-adapted vectors for the expression of tagged proteins in mycobacteria for use in formaldehyde crosslinking procedures to identify protein–protein interactions in mycobacteria.

## 2 Materials and methods

### 2.1 Bacterial strains and growth conditions

For *in vivo* protein cross-linking experiments we used *Mycobacterium smegmatis groEL1ΔC* (Noens et al., 2011), which was transformed with the appropriate plasmids and grown in LB medium (5 g yeast extract, 5 g NaCl, 10 g Tryptone per liter) with continuous shaking (150 rpm) at 37 °C. 0.05% (v/v) Tween 80 was added to LB to avoid clumping.

### 2.2 Construction of Gateway adapted vectors for expression of tagged protein expression in mycobacteria

**Fig. 1:**
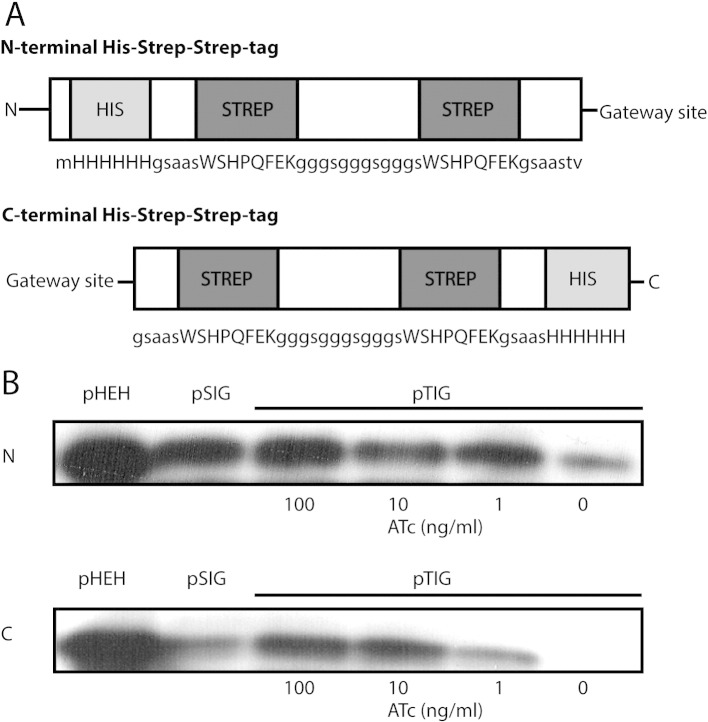
Expression vector construction. (A) Sequence information for the expression plasmids. pHEH-N, pSIG-N and pTIG-N contain an N-terminal His–Strep–Strep tag linked to the Gateway insertion site by a flexible glycine-rich linker. pHEH-C, pSIG-C, and pTIG-C are C-terminal versions of these same vectors. Complementary oligonucleotides were annealed to produce the Strep-II tag sequence plus glycine linkers and PCR used to introduce a ribosome binding site, his-tag and restriction sites. (B) Expression from the crosslinking vectors determined by Western blotting. *M. smegmatis* expressing the control protein Rv1636 (15 kDa) from the six expression vectors was grown in LB + 0.05% Tween 80 for 24 h, in the presence of a range of anhydrotetracycline (ATc) concentrations from 0 to 100 ng/ml for the pTIG vectors. The upper and lower panels show expression from N-terminal and C-terminal tagging vectors respectively. Cells were lysed, and expression of the tagged proteins detected by Western blotting.

**Fig. 2:**
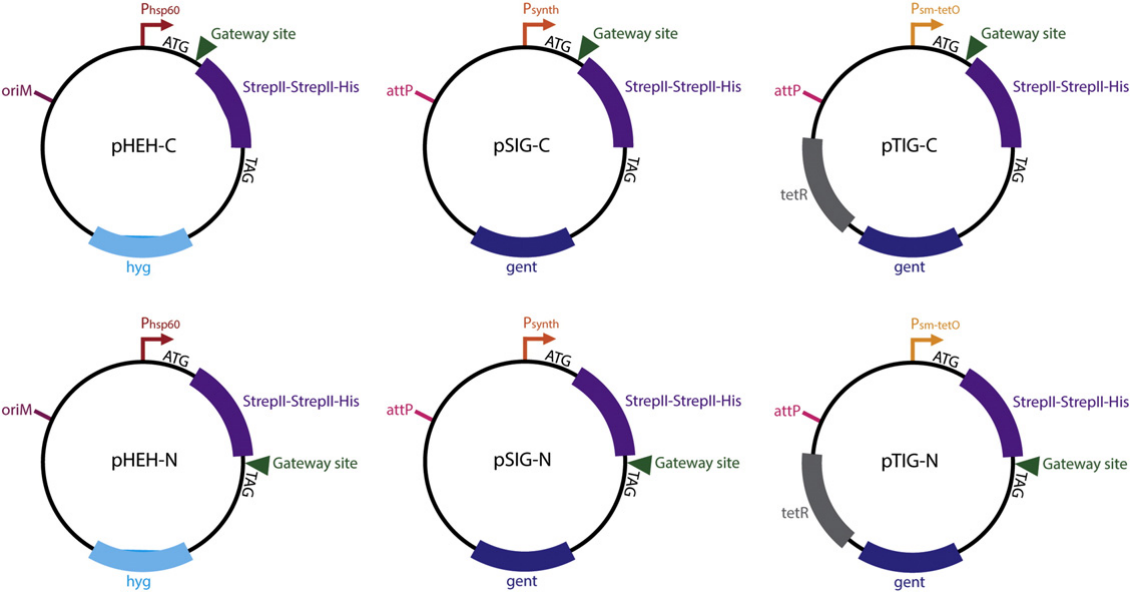
Schematics of the six Gateway adapted vectors constructed and described in this study.

Complementary 108 base pair oligonucleotides containing two Strep-II tags separated and flanked by flexible glycine linkers (GGCAGCGCCGCGAGCTGGAGCCACCCGCAGTTCGAGAAGGG CGGTGGCAGCGGCGGTGGCAGCGGCGGTAGCTGGAGCCACCCGCAGTTCGAGAAGGGCAGCGCCGCG) were annealed by heating to 95 °C and cooling slowly to room temperature in the heat block. The annealed oligonucleotides were used as a PCR template to construct the final tags. Primers were designed to amplify the Strep-II tags with the addition of a His-tag, a ribosome binding site and a C- or N-terminal ScaI restriction site (Cterm_F: TTAATTAACCGGAGGAATCACTTCGCAATGGGCCGGCGAAGTACTGGGCAGC GCCGCGAGCTGGAGC, Cterm_R: CGTACGATCGATTCAATGGTGGTGGTGGTGGTGCGCG GCGCTGCCCTTCTCGAA, Nterm_F: TTAATTAACCGGAGGAATCACTTCGCAATGGGCCGG CGACACCACCACCACCACCATGGCAGCGCCGCGAGCTGGAGC, Nterm_R: CGTACGATCG ATTCACAGTACTCGCGGCGCTGCCCTTCTCGAA). The PCR products were cloned into pTETR2 *via* the PacI and BsiWI sites. pTETR2 is an integrating vector containing a strong mycobacterial promoter into which *tet* operator sequences have been inserted along with a *tetR* gene encoding a repressor which binds the operator sequences and occludes ribosome binding in the absence of anhydrotetracycline (Schuessler et al., 2013). The vectors were then Gateway adapted *via* the ScaI site using the RfC.1 fragment (Gateway Vector Conversion System, Invitrogen), producing pTIG-N and pTIG-C. The inserts were subcloned into pMV762, a hygromycin resistant episomal plasmid containing the *hsp60* promoter based on pMV261 (Stover et al., 1991), to produce pHEH-N and pHEH-C, and into an integrating vector containing an intermediate synthetic promoter to produce pSIG-N and pSIG-C. Sequence information for the plasmids and plasmid schematics is shown in Fig. 1, Fig. 2

### 2.3 Formaldehyde crosslinking and purification of protein complexes

We first optimized the concentration of formaldehyde and the incubation time required to induce sufficient crosslinking while minimizing non-specific complex formation. A range of concentrations from 0.2 to 2% formaldehyde were added to cultures and incubated for 10 and 20 min with gentle shaking. The formaldehyde was quenched by the addition of 1/10 culture volume of ice cold glycine in PBS (0.125 M final glycine concentration). Cell lysates were prepared by vortexing for 2 min with an equal volume of 0.2 μm glass beads. To prepare the proteins for separation by SDS-PAGE without reversing the formaldehyde crosslinking, the lysates were heated at 65 °C for 20 min as described in Klockenbusch and Kast (2010). Following SDS-PAGE, the complexes were visualized by Western blotting with an HRP-conjugated penta-His antibody (Qiagen) to identify the lowest concentration of formaldehyde that led to a shift of the AceE band to a higher molecular weight, indicating that it was found in complexes rather than as a monomer. Following establishment of an optimized protocol, *M. smegmatis* was grown in 2 L of LB-Tween 80 medium until stationary phase when the culture was used for *in vivo* crosslinking experiments. Formaldehyde at a final concentration of 0.4% was added to the culture and incubated at room temperature for 10 min before quenching with PBS–glycine. The culture was harvested at 10,000 ×*g* for 10 min, the pellets washed once in PBS, then resuspended in 20 ml 50 mM Tris–HCl (pH8) and 150 mM NaCl. The cells were incubated at room temperature for 20 min with 2.4 mg/ml lysozyme then sonicated at 40% amplitude on ice for 10 min, with a 2 second pulse followed by a 2 second pause. The Strep/His-tagged complexes were purified using a two-step procedure of StrepTactin FPLC followed by nickel affinity purification. Lysates were cleared by centrifugation at 14,000 ×*g* for 45 min, loaded onto a StrepTactin column and washed with 50 mM Tris–HCl (pH8) and 150 mM NaCl until the baseline UV absorption reading returned to zero. The proteins were eluted with 2.5 mM desthiobiotin, with a 1 hour pause used to release tightly binding complexes. Pooled fractions were loaded onto a nickel-affinity column in the presence of 40 mM imidazole and washed at 100 mM imidazole in 50 mM Tris–HCl (pH8) and 150 mM NaCl to remove any non-specific contamination. Complexes were eluted with 500 mM imidazole and pooled fractions concentrated using an Amicon 15 kDa cut-off centrifugation device.

### 2.4 In gel tryptic digestion

The crosslinking of the purified complexes was reversed by boiling in Laemmli buffer for 20 min and the proteins separated by SDS-PAGE. After running the proteins on an SDS PAGE gel, staining with Imperial Blue (Thermo) and destaining, bands were cut out of the gel and added to 0.5 ml silated microfuge tubes. 200 μl 50 mM ammonium bicarbonate pH 8.4 was added followed by 200 μl of acetonitrile and incubated at room temperature for 5 min. The supernatant was removed and the process repeated until no further color remained in the gel pieces, and the gel pieces dried in a SpeedVac for 15 min. The reduction and carboxymethylation of the proteins were achieved by adding 20 μl 10 mM DTT solution and incubating for 60 min at 57 °C. The DTT was removed and the gel pieces rinsed briefly with acetonitrile, 20 μl 55 mM iodoacetamide solution added, and incubated for 30–45 min in the dark at room temperature. The iodoacetamide solution was removed and the gel pieces washed with 500 μl of 50 mM ammonium bicarbonate pH 8.4 for 15 min, which was then removed and 200 μl acetonitrile added for 5 min. The gel pieces were dried by washing twice with 200 μl acetonitrile and the acetonitrile removed. For tryptic digestion, 20 μl trypsin solution was added to gel pieces and incubated for 15–20 min at room temperature and then further ammonium bicarbonate solution was added to cover the gel pieces, if needed, and incubated at 37 °C overnight. To elute the peptides from the gel pieces, the peptide solution was removed into a clean silated tube. The gel pieces were washed with 50% acetonitrile, adding sufficient to cover the gel pieces and incubated at 37 °C for 15 min. This wash was added to the saved supernatant, the volume reduced to 1–2 μl in a SpeedVac, 30 μl 1% acetonitrile added and acidified with 0.1% formic acid, and the sample frozen − 20 °C. Prior to MS the sample was spun at 10,000 ×*g* for 10 min and the supernatant removed for MS.

### 2.5 Peptide analysis using high pressure liquid chromatography and mass spectrometry

Samples were loaded on a Zorbax SB-C18 5µm, 35 x 0.5 mm (Agilent) trap column and washed for 60 min using 96.7% water: 3% acetonitrile: 0.3% formic acid; the extended wash was to remove residual KCl remaining from the ion exchange purification step. Peptides were separated using a Zorbax 300SB-C18 5µm, 150 x 0.3 mm capillary column (Agilent) at a flow rate of 5 ml/min using an Agilent 1100 HPLC system. Buffer C (94.9% water: 5% acetonitrile: 0.1% formic acid) and Buffer D (94.9% acetonitrile: 5% water: 0.1% formic acid) were used for the elution of peptides according to the following program: gradient 0–30% Buffer D over 90 min, gradient 30–90% Buffer D over 10 min, 90% Buffer D for 10 min, gradient 90–100% Buffer D over 1 min and 100% Buffer D for 10 min. Eluted peptides were analyzed using a Q-TRAP mass spectrometer (Applied Biosystems) equipped with a Turbo Spray Ion source at 150 μC. Data were collected with an IDA method consisting of a survey scan (350 m/z to 1200 m/z), an enhanced resolution scan and four enhanced product ion scans. Dynamic background subtraction was used prior to ion selection; the four most abundant doubly or triply charged ions were selected for the product ion scans. The resulting spectra were analyzed and quantified using ProteinPilot software (Applied Biosystems), quoted significance values were obtained with the inbuilt statistical analysis tool.

## 3 Results and discussion

The analysis of protein–protein interactions in mycobacteria has the potential to shed light on the functions of the large number of predicted open-reading frames annotated as conserved hypothetical proteins. We have developed a formaldehyde crosslinking system to detect *in vivo* interactions in mycobacteria.

We set out to construct a series of Gateway-adapted vectors for the expression of tagged proteins in mycobacteria. To construct the expression vectors, we used three promoter strengths: the strong constitutive *hsp60* promoter, an intermediate synthetic promoter (E. O. Davis, unpublished), and a titratable anhydrotetracycline-inducible promoter (Schuessler et al., 2013). We chose to use a combination of either an N- or C-terminal tandem Strep-tag and a His-tag. The His-tag has the advantage that it can be used to purify proteins under denaturing conditions, allowing the analysis of interactions with difficult-to-purify proteins such as those found in the cell wall. We expressed proteins in an *M. smegmatis* strain that had been engineered to remove a native 5-His moiety in the GroEL chaperone that has previously interfered with His-tag purification from this species (Noens et al., 2011). *M. smegmatis* has the advantage of being easier to handle than the category III *M. tuberculosis*. It should be noted that the vectors designed for these experiments are also functional in *M. tuberculosis*. Gateway-adaptation of the vectors allows for quick, cloning-free generation of expression constructs using a recombination-based approach (Hartley et al., 2000). Sequence information for the plasmids and plasmid schematics is shown in Fig. 1, Fig. 2.

To validate the system, we used the AceE component of the pyruvate dehydrogenase complex—a large complex comprised of three subunits (E1, E2 and E3). In *M. tuberculosis*, these subunits are E1:AceE (Rv2241), E2:DlaT (Rv2215) and E3:LpdC (Rv0462). In *M. smegmatis*, their homologues are MSMEG_4323, SucB (MSMEG_4283) and LpdA (MSMEG_0903). The *aceE* (Rv2241) gene from *M. tuberculosis* was amplified and inserted into pENTR/D/TOPO (Invitrogen) to construct an entry vector that can be used with any of the destination vectors described above. The six expression vectors were transformed into *M. smegmatis*, with co-transformation of pBS-Int (Springer et al., 2001) in the case of the pSIG and pTIG vectors, to provide the integrase gene *in trans* to facilitate chromosomal integration. These vectors lack an integrase gene to reduce loss of these plasmids once integration into the genome has taken place.

The construction of the six vectors was carried out to allow a range of expression levels to be tested and an N- or C-terminal tag to be used to achieve optimal expression of the target protein. In the case of AceE, it was observed that C-terminal tagged versions of the protein are not well-expressed, and the high expression levels generated from the *hsp60* promoter vectors led to the reduced growth rates of the cells, making these vectors unsuitable for use in the crosslinking experiments. Therefore, to test expression from the six vectors in parallel we used as a control the *Mtb* protein Rv1636, which was chosen as its overexpression has not been shown to be toxic to *M. smegmatis* and as shown in Fig. 1B it was successfully expressed from each of the 6 vectors.

To test the effectiveness of *in vivo* crosslinking with these vectors, a pTIG-N (AceE) construct, which drives expression of N-terminally tagged AceE from an anhydrotetracycline-inducible promoter, was chosen. Expression was induced with 100 ng/ml ATc, as this concentration was well-tolerated by the cells and generated enough protein to allow purification of crosslinked complexes. We optimized the concentration of formaldehyde and the incubation time required to induce sufficient crosslinking while minimizing non-specific complex formation as described in the Materials and methods section. The complexes were visualized by Western blotting for the His-tag to identify the lowest concentration of formaldehyde that led to a shift of the AceE band to a higher molecular weight, indicating that it was found in complexes rather than as a monomer, and we settled on 0.4% formaldehyde for AceE experiments. We suggest that this optimization step is performed for each protein of interest.

**Fig. 3:**
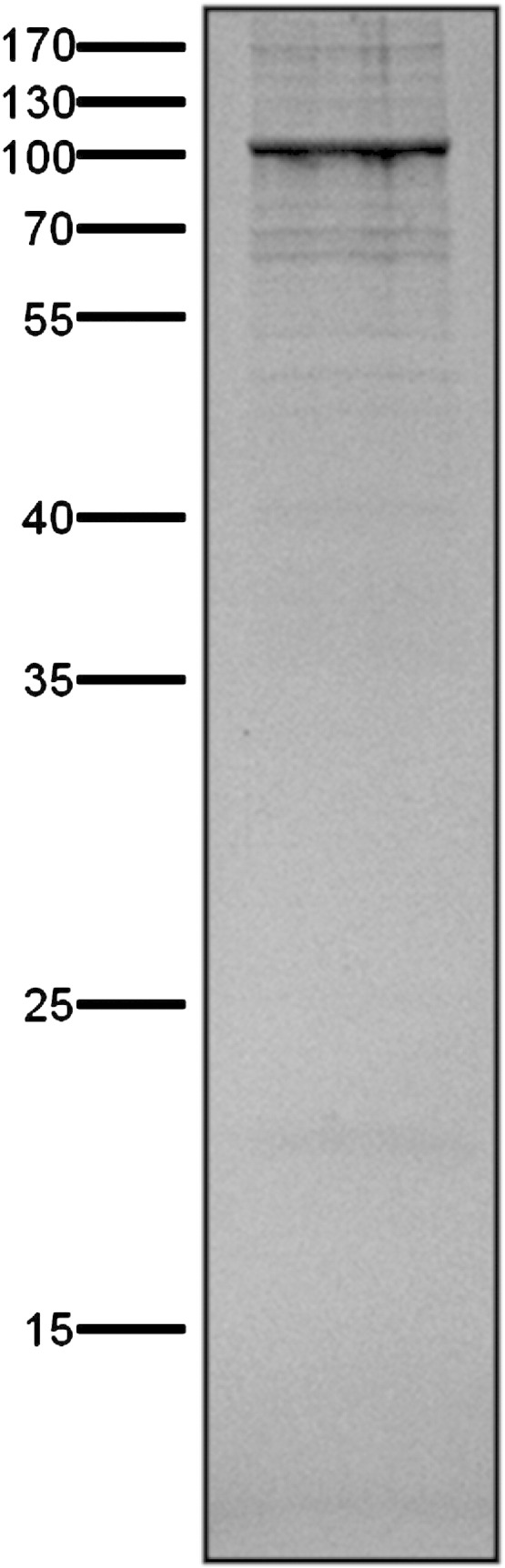
AceE interacting proteins. *M. smegmatis* containing pTIG-N::aceE grown in LB + 0.05% Tween 80 was induced with 100 ng/ml ATc for 24 h and complexes crosslinked with 0.4% formaldehyde. The Strep/His-tagged complexes were purified as described in the Materials and Methods section, crosslinks were reversed by heating at 95 °C for 20 min and the proteins separated by SDS-PAGE. A control of wild type cells yielded no complexes and is not shown.

To identify interactions with AceE, we grew 1 liter cultures of the pTIG-N (AceE) containing *M. smegmatis* strain and a control strain without the plasmid in LB + 0.05% Tween 80 for 24 h and formaldehyde cross-linked protein complexes were purified from the resulting cell free extracts by sequential purification on StrepTactin and nickel-affinity columns. The crosslinking of the purified complexes was reversed by boiling in Laemmli buffer and the proteins separated by SDS-PAGE. Fig. 3 shows the SDS-PAGE analysis of the eluted proteins. No proteins were purified from the control culture (not shown) whereas a number of bands were co-purified with AceE. The identity of the bands following trypsin digestion and LC–MS is shown in Table 1 (Trauner et al., 2011). Of the interactions detected, two were the expected DlaT and LpdC components of the pyruvate dehydrogenase complex, indicating that this method is capable of accurately detecting protein interactions in mycobacteria. In addition, several proteins not previously identified as interacting partners for pyruvate dehydrogenase were also identified and it remains to be determined what role these interactions play *in vivo*. It should, however, be noted that there are a number of biotinylated proteins in mycobacteria, including some of those co-purified with AceE. Biotinylated proteins will bind the StrepTactin with a high affinity and the possibility of these interactions being false positives should be considered. Furthermore, the co-purification of highly abundant proteins such as stress proteins or components of the ribosome may also be due to non-specific binding. For proteins which associate with highly promiscuous proteins such as chaperones, it may be advantageous to narrow down the possible interactors by excluding the crosslinking step.

**Table 1:** Identification of AceE interactors. LC–MS data were extracted and files from each band analyzed independently and as a pooled set. Proteins identified with at least 2 unique peptides were retained.

Accession	Gene name	Description	Unique peptides
Q10504	*aceE* (Rv2241)	*M. tuberculosis* AceE component (bait)	31
A0R0B0	MSMEG_4323	Pyruvate dehydrogenase E1 component (AceE homologue)	22
A0QQW8	*lpdA* (MSMEG_0903)	Dihydrolipoyl dehydrogenase (LpdC homologue)	5
A0R072	*sucB* (MSMEG_4283)	2-Oxoglutarate dehydrogenase, E2 component, dihydrolipoamide succinyltransferase (DlaT homologue)	3
A0QTE1	MSMEG_1807	Acetyl-/propionyl-coenzyme A carboxylase alpha chain^a^	21
A0QS98	*Tuf*	Elongation factor Tu	12
A0QQC8	*dnaK*	Chaperone protein DnaK	9
A4ZHR8	MSMEG1842	Adenosylhomocysteinase	3
A0QTE7	MSMEG_1813	Propionyl-CoA carboxylase beta chain^a^	2
A0R616	MSMEG_6391	Propionyl-CoA carboxylase beta chain^a^	2

a Known biotinylated proteins.

We believe that our system for the expression of tagged target proteins in mycobacteria combined with *in vivo* crosslinking is suitable for purifying protein complexes with no or little detectable background. This method allows for the identification of weak or transient interactions while allowing sufficient purification of complexes to reduce false positives. The study of protein interactions in mycobacteria rather than surrogate hosts such as *Escherichia coli* has the advantage that the cellular environment is conducive to real interactions taking place rather than the false positives or false negatives common with other hosts. The study of large complexes is also difficult in other hosts as interacting pairs of proteins may need additional factors or proteins to be present for an interaction to occur. One weakness of this crosslinking approach that we can anticipate is that the high levels of protein overexpression under non-native conditions could lead to the identification of false positives. For this reason, we also generated regulatable version of the vectors to allow expression to be induced at lower levels and under biologically relevant conditions.

Our system has proven itself to be capable of detecting known protein interactions and we hope that it will prove useful for the study of unknown proteins in mycobacteria. Combined with independent methods of investigating interactions, it will be possible to minimize false positives and tease apart essential virulence pathways in *Mtb*.

## 4 Concluding remarks

We have developed Gateway adapted vectors which allow the overexpression of Strep–Strep–His-tagged proteins from promoters of different strengths in mycobacteria and show that they are suitable for use in *in vivo* crosslinking experiments.
